# Expression and prognostic value of JAM-A in gliomas

**DOI:** 10.1007/s11060-017-2555-0

**Published:** 2017-07-04

**Authors:** Ann Mari Rosager, Mia D. Sørensen, Rikke H. Dahlrot, Henning B. Boldt, Steinbjørn Hansen, Justin D. Lathia, Bjarne W. Kristensen

**Affiliations:** 1 Department of Pathology, Odense University Hospital, Winsløwparken 15, 3rd floor, 5000 Odense, Denmark; 2 Department of Clinical Research, University of Southern Denmark, Winsløwparken 19, 3rd floor, 5000 Odense, Denmark; 3 Department of Oncology, Odense University Hospital, Sdr. Boulevard 29, 5000 Odense, Denmark; 4 Department of Cellular and Molecular Medicine, Lerner Research Institute, 9500 Euclid Avenue, NC10, Cleveland, OH 44195 USA

**Keywords:** Astrocytic brain tumors, Glioma, Junctional adhesion molecule-A, Tumor stem cell, Prognosis

## Abstract

Gliomas are among the most lethal cancers, being highly resistant to both chemo- and radiotherapy. The expression of junctional adhesion molecule-A (JAM-A) was recently identified on the surface of stem cell-like brain tumor-initiating cells and suggested to function as a unique glioblastoma niche adhesion factor influencing the tumorigenic potential of brain tumor-initiating cells. We have recently identified high JAM-A expression to be associated with poor outcome in glioblastomas, and our aim was to further investigate the expression of JAM-A in gliomas focusing especially on the prognostic value in WHO grade II and III gliomas. JAM-A protein expression was evaluated by immunohistochemistry and advanced quantitative image analysis with continuous estimates of staining intensity. The JAM-A antibody stained tumor cell membranes and cytoplasm to various extent in different glioma subtypes, and the intensity was higher in glioblastomas than low-grade gliomas. We could not detect an association with overall survival in patients with grade II and III tumors. Double-immunofluorescence stainings in glioblastomas revealed co-expression of JAM-A with CD133, SOX2, nestin, and GFAP in tumor cells as well as some co-expression with the microglial/macrophage marker IBA-1. In conclusion, JAM-A expression was higher in glioblastomas compared to low-grade gliomas and co-localized with recognized stem cell markers suggesting an association of JAM-A with glioma aggressiveness. No significant association between JAM-A expression and overall survival was found in grade II and III gliomas. Further research is needed to determine the function and clinical impact of JAM-A in gliomas.

**Electronic supplementary material:**

The online version of this article (doi:10.1007/s11060-017-2555-0) contains supplementary material, which is available to authorized users.

## Introduction

Gliomas are the most frequent type of primary tumors in the central nervous system (CNS) [1]. The aggressiveness of gliomas has been suggested to be associated with brain tumor-initiating cells (BTICs) that have the ability to self-renew and give rise to new tumors [2–4]. BTICs are relatively treatment-resistant and are thought to be located mainly in perivascular and hypoxic niches [5–8]. These niches may be maintained by adhesion molecules e.g., integrin-α6 and laminin-α2 [8, 9]. Junctional adhesion molecule-A (JAM-A, also known as JAM-1) is a transmembrane protein with both extra- and intracellular domains belonging to the immunoglobulin superfamily [10–13]. Using high-throughput flow cytometry screening, JAM-A has been found to be a glioblastoma (GBM) niche adhesion factor influencing the tumorigenic potential of BTICs [14]. In a follow-up study, it was reported that JAM-A overexpression could drive self-renewal and was suppressed by microRNA-145 [15]. Messenger RNA data from the National Cancer Institute REpository for Molecular BRAin Neoplastic Data (NCI REMBRANDT) suggested that high levels of JAM-A are associated with poor outcome in glioma patients, and we recently demonstrated that high JAM-A protein expression is associated with shorter survival in patients with GBM [14]. JAM-A was initially identified on platelets and later on endothelial and epithelial cells [16, 17] and has been associated with different functions like monocyte/leukocyte transmigration [10, 17–19], but its function in cancer remains unclear, as both high and low expression levels have been associated with poor prognosis. In lung and nasopharyngeal carcinomas, high expression has been correlated with poor prognosis [20, 21], but in kidney, pancreatic, and gastric cancer high expression is associated with a better outcome [22–24]. In breast carcinomas, both high and low expression levels have been correlated with poor outcome, most likely due to the selection of patients with different tumor types [25–27]. Functional studies in triple-negative breast cancer cells demonstrated that JAM-A was necessary and sufficient for self-renewal [28].

The aim of the present study was to investigate the expression and prognostic value of JAM-A in two glioma patient cohorts using immunohistochemistry and advanced quantitative image analysis. The Region of Southern Denmark (RSD) glioma cohort is population-based and includes astrocytic and oligodendroglial tumors, whereas the Odense University Hospital (OUH) cohort contains astrocytic tumors. First, we focused on expression and prognostic value of JAM-A in World Health Organization (WHO) grade II or III gliomas using the RSD cohort, and then on its expression and prognostic value in patients with diffuse (DA) and anaplastic astrocytoma (AA) using the OUH cohort. The expression of JAM-A was assessed using advanced automated image analysis, a method previously used by our group to investigate biomarkers resulting in continuous measurements [29–33]. To explore the association of JAM-A with stemness, a double-immunofluorescence panel was established consisting of BTIC markers: CD133 [34, 35], SOX-2 [36, 37], and nestin [38, 39], an astrocytic marker: glial fibrillary acidic protein (GFAP), and a microglial/macrophage marker: ionized calcium-binding adapter molecule-1 (IBA-1). Being highly malignant and containing the highest frequency of BTICs, this was investigated in GBMs.

## Materials and methods

### Tissue samples

The RSD glioma cohort consists of 433 patients diagnosed with primary gliomas between 01.01.2005 and 31.12.2009. Of these, 43 patients with WHO grade II and III gliomas had a sufficient amount of viable tumor tissue available for JAM-A immunohistochemical analysis including patients with DA (n = 11), oligodendroglioma (OD) (n = 11), AA (n = 16), and anaplastic oligodendroglioma (AOD) (n = 5). The cohort is well-characterized and has been used in other studies [29–31, 40, 41].

The OUH astrocytoma cohort consists of 111 patients diagnosed with primary astrocytic tumors between 1994 and 2005. Of these, 32 patients with DA (n = 21) and AA (n = 11) had a sufficient amount of viable tumor tissue available for JAM-A immunohistochemical analysis. The OUH astrocytoma cohort has been used for previous biomarker studies [33, 42].

For both cohorts, no treatment was received prior to surgical resection. All tumor samples were reclassified according to the 2016 WHO classification [1]. Patient characteristics are shown in Table 1.

**Table 1:** Clinicopathological characteristics

	RSD glioma cohort		OUH astrocytoma cohort	
	WHO II	WHO III	WHO II	WHO III
Number of patients	22	21	21	11
Median survival (months)	65.2	28.2	57.1	14.1
Age (median)	38.5	53.6	45.1	50.7
Sex (male/female)	13/9	17/4	14/7	7/4
Performance status (0–1/2–4)	16/6	18/3	n.d	n.d
Endpoint (alive/dead)	8/14	2/19	5/16	0/11
IDH status (mutated/wildtype)	20/2	15/6	12/9	2/9
ATRX status (retained/loss)	13/9	10/11	6/15	7/4
P53 status (positive/negative)	8/14	13/8	8/13	3/8
Subtype (astrocytic/oligodendroglial^a^)	11/11	16/5	21/0	11/0

*n.d.* not determined, *OUH* Odense University Hospital astrocytoma cohort, *RSD* Region of Southern Denmark glioma cohort

^a ^Oligodendroglial tumors were diagnosed based on the 2016 WHO classification and thus defined as tumors with IDH mutation and 1p19q co-deletion

Normal brain tissue specimens were obtained from two adult patients at autopsy. Cause of death was not related to diseases in the CNS.

This study was approved by the local Committee on Health Research Ethics and the Danish Data Protection Agency. Use of tissue was not precluded by any patients according to Danish Tissue Application Register.

### Immunohistochemical staining

Fresh tissue was fixed in 4% neutral buffered formaldehyde and paraffin-embedded. Three micrometer sections were cut on a microtome and stained routinely with haematoxylin-eosin to define representative tumor regions.

Paraffin sections were stained on a Dako Autostainer Universal Staining System (Dako, Denmark). The sections were deparaffinized, and heat-induced epitope retrieval (HIER) was performed by incubation in a buffer solution consisting of 10 mmol/L Tris-base and 0.5 mmol/L ethylene glycol tetraacetic acid, pH 9. After blocking of endogenous peroxidase activity with 5% hydrogen peroxide, the sections were incubated for 60 min with primary antibody against JAM-A/F11R (2E3-1C8, 1 + 400, Sigma-Aldrich, USA). The same antibody clone was used for both cohorts. Detection and visualization of antigen–antibody complex was performed using PowerVision (Novocastra, United Kingdom) and diaminobenzidine (DAB) as chromogen, respectively. Finally, sections were counterstained with Mayers Haematoxylin. Omitting primary antibody and isotype control served as negative controls as well as controls for non-specific staining related to the detection system (Online Resource 1). A tissue microarray consisting of nine different GBMs as well as normal colon, cerebellum, placenta, and rat hippocampus was used as positive/negative control and to monitor inter-run staining variation.

### Quantification

Slides were scanned on a Digital Slide Scanner (Hamamatsu Photonics, Japan). The JAM-A staining was analyzed using the Tissuemorph module in the software program Visiopharm Integrated System (Visiopharm, Denmark). Sample images were collected using systemic uniform random sampling (meander fraction-based). Sampling was performed at 20× magnification with a sample fraction of 10% as previously described [29]. Images were reviewed ensuring high image quality and sampling of vital tumor tissue only. Images were excluded according to the following criteria: less than 50% vital tumor tissue due to presence of normal brain tissue, infiltration zones, and necrotic areas as well as substantial non-specific background staining and staining artifacts. Blood vessels were removed manually in each image. Five tumors had less than five images available and were resampled with a sample fraction of 30%.

Pixel-based software classifiers were trained based on nuclear identification. The cytoplasm/membrane was identified in a radius of three micrometers from the nucleus as previously described [29, 33]. The classifier labeled the nucleus with green and the perinuclear area with light blue. The classifier was trained on different types of gliomas to take the heterogeneity of gliomas into account. The mean intensity of the perinuclear light blue area of all identified cells per tumor was measured resulting in a mean estimate of the JAM-A staining intensity.

### Detection of isocitrate dehydrogenase (IDH) mutations

Sections from all patients included in the two cohorts were stained with an antibody against the most common IDH mutation IDH1-R132H (mIDH1R132H, clone H14, 1:100, Dianova, Germany) using the BenchMark Ultra IHC/ISH staining system (Ventana Medical Systems Inc, USA) as previously reported [40, 41]. When no IDH1-R132H mutation was detected immunohistochemically, next generation sequencing (NGS) was performed to detect other mutations in the IDH1/2 genes. The gene panel used included 20 glioma-associated genes and is described in detail by Zacher et al. [43]. NGS libraries and analyses were done as previously reported [43].

### Detection of ATRX and p53

Nuclear expressions of a-thalassemia/mental retardation X-linked syndrome (ATRX) and p53 were demonstrated immunohistochemically using a rabbit polyclonal antibody (HPA001906, 1:100, Atlas Antibodies, Sweden) and a monoclonal antibody (DO7, Ready-to-use, Ventana Medical Systems Inc), respectively. The two immunohistochemical staining protocols were performed for all tumors using the BenchMark Ultra IHC/ISH staining system (Ventana Medical Systems Inc).

### Detection of 1p19q deletions

Testing for co-deletion of chromosomal arms 1p19q was performed on all tumors that showed retained nuclear expression of ATRX. 1p19q status was determined by fluorescence in situ hybridization (FISH) on formalin-fixed paraffin embedded tumor tissue using the Vysis LSI 1p36/LSI 1q25 and LSI 19q13/19p13 Dual-Color Probe (Abbott Molecular, Vysis, USA). The FISH procedure was performed using the Dako Histology FISH Accessory Kit K5799. For some tumors, FISH analysis was inconclusive, and 1p19q status was determined by accessing copy number variation of chromosomes 1 and 19 using the Infinium Methylation 850K EPIC BeadChip array (Illumina, USA) according to manufacturer’s description.

### Double-immunofluorescence

Double-immunofluorescence was performed on tissue microarray containing nine GBMs. The preparations as well as the first step in the stainings are as described above. After detection of JAM-A (1 + 200) using Catalyzed Signal Amplification II kit with FITC (CSA II, Dako), sections were incubated with antibodies against nestin (196,908, 1 + 200, R&D systems, USA), CD133 (W6B3C1, 1 + 40, Miltenyi Biotec, Germany), GFAP (Z0334, 1 + 8000, Dako), SOX-2 (245,610, 1 + 400, R&D systems), or IBA-1 (019-19741, 1 + 4000, Wako Pure Chemical Industries, Japan) followed by Tyramide Amplification Signal Cyanine-5 (TSA-Cy5, Perkin Elmer, USA). Nuclei were counterstained with 4.6-diamidino-2-phenylindole (DAPI) (VWR International, USA). Fluorescence images were taken with a Leica DM6000B microscope connected to an Olympus DP72 1.4 Mega Pixel CCD (Olympus, Japan) camera using DAPI (Omega XF06, Omega Optical, USA), FITC (Leica, Germany), and Cyanine-5 (Omega XF110-2) filters. Due to cross-reaction, a different JAM-A antibody-clone (EP1042Y, 1 + 400, Abcam, United Kingdom) was used for the double staining with CD133.

### The Cancer Genome Atlas (TCGA)

mRNA expression levels of JAM-A (F11R) in primary, secondary, and recurrent GBMs were investigated using GlioVis (https://gliovis.bioinfo.cnio.es). Data were available for 497 primary, 7 secondary, and 16 recurrent GBMs, and the dataset was exported directly from GlioVis [44].

### Statistical analysis

Comparison of JAM-A intensity among tumor types and grades was done using the one-way analysis of variance followed by Bonferroni’s multiple comparison test or Student’s unpaired *t*-test. JAM-A intensity data for normal brain and GBMs from the RSD cohort have been published earlier [14], but was included for comparison with grade II and III gliomas. The univariate relationships were illustrated by Kaplan–Meier plots and differences in overall survival (OS) compared using log-rank test. The median JAM-A intensity was used as a pre-specified cut-off value in the survival analyses. Multivariate Cox proportional hazard regression analyses were performed for patients with grade II and III tumors separately. All assumptions were tested, and all analyses were carried out in STATA.

Patients were followed until death; patients still alive were censored in May 2017 for the RSD glioma cohort and April 2017 for OUH astrocytoma cohort. OS was defined as time from primary surgery until death or censoring.

## Results

### JAM-A expression in normal brain

**Fig. 1:**
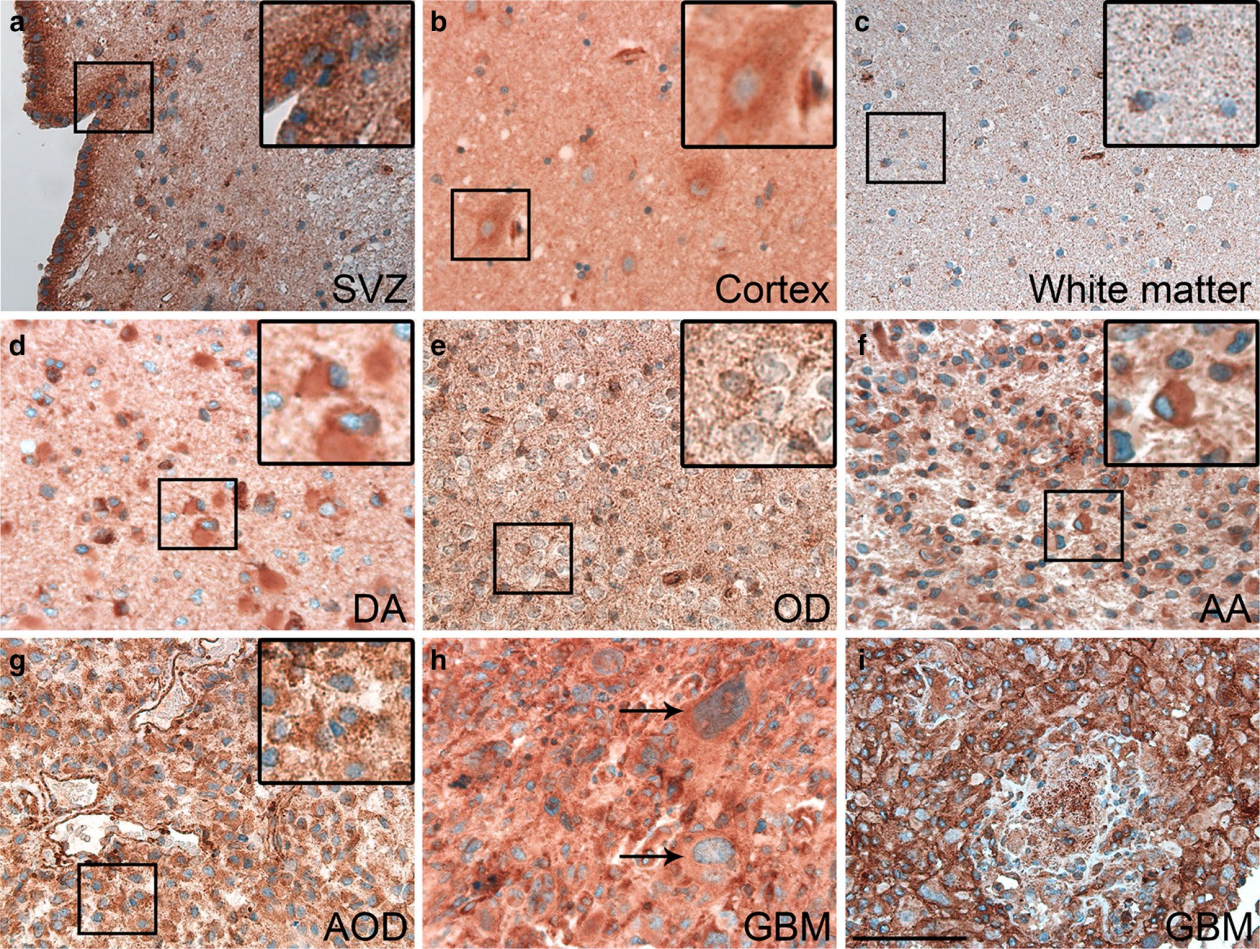
Examples of JAM-A staining in normal brain and WHO grade II-IV gliomas, immunohistochemically stained with JAM-A antibody. **a** Subventricular zone (SVZ) with positive ependymal layer. **b** Weak neuronal staining was seen in cortex. **c** Few positive cells were observed in white matter. **d** Diffuse astrocytoma (DA) with moderate staining showing positive gemistocytes. **e** Oligodendroglioma (OD) with moderate staining intensity. **f** and **g** Anaplastic astrocytoma (AA) and anaplastic oligodendroglioma (AOD) with moderate staining intensity. **h** and **i** Glioblastoma (GBM) with giant cells showing moderate staining intensity, and glomeruloid vessels with staining of the endothelium as well as stained cells with tumor cell morphology. *Scale bar* 100 µm

In normal brain tissue, the ependymal layer of the ventricles expressed JAM-A, and a few cells below the ependymal layer were positive (Fig. 1a). Neurons in the neocortex were positive (Fig. 1b), and the neuropil showed weak positivity (Fig. 1b). Only a few positive cells were identified in the white matter (Fig. 1c). The endothelium in blood vessels was positive, whereas the muscular layer was negative.

### JAM-A expression in gliomas

The overall histological pattern revealed a weak to intense staining of a low to high fraction of the tumor cells. JAM-A was expressed in the cytoplasm and the membrane of tumor cells in all gliomas (Fig. 1d–i). For grade II gliomas, different staining patterns were observed. Some had a weak staining, while others had intense staining. This was the case for DAs with gemistocytic tumor cells (Fig. 1d) as well as ODs with small oligodendrocyte-like tumor cells (Fig. 1e).

Both AAs (Fig. 1f) and AODs (Fig. 1g) showed weak to moderate JAM-A positivity, while most GBMs had moderate to intense JAM-A expression including glomeruloid tufts (Fig. 1h, i). In the infiltration zones, small positive cells as well as positive neurons were noticed (not shown).

### JAM-A and tumor grade

**Fig. 2:**
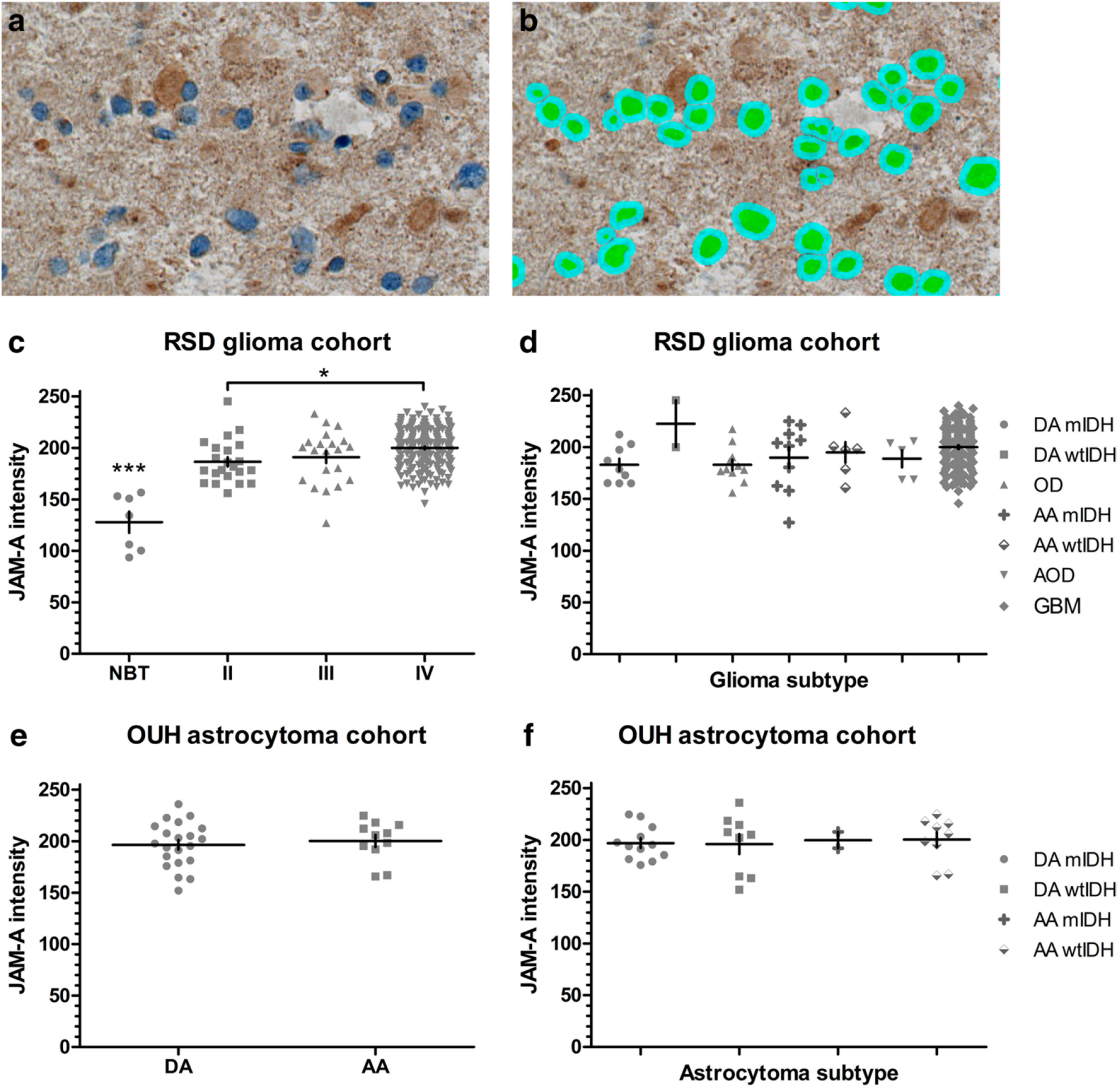
Association of JAM-A intensity with tumor type. Using immunohistochemical staining JAM-A+ tumor cells were identified. **a** and **b** When the original image was processed and the algorithm applied, nuclei of JAM-A+ cells were represented by *green* and perinuclear areas by *light blue*. The staining intensity was measured in the perinuclear area. **c** In the RSD glioma cohort, JAM-A intensity increased with tumor grade and was higher in gliomas compared to normal brain tissue. **d** No difference was seen among the different types of gliomas in the RSD glioma cohort. **e** and **f** In the OUH astrocytoma cohort, JAM-A intensity in DAs and AAs did not differ significantly from each other, and similar was found when subdividing the tumors based on IDH status. *p-value < 0.05, ***p-value < 0.001. The *vertical lines* indicate mean +/− standard error of the mean. *AA* anaplastic astrocytoma, *AOD* anaplastic oligodendroglioma, *DA* diffuse astrocytoma, *GBM* glioblastoma, *mIDH* mutated isocitrate dehydrogenase, *NBT* normal brain tissue, *OD* oligodendroglioma, *OUH* Odense University Hospital, *RSD* Region of Southern Denmark, *wtIDH* wildtype isocitrate dehydrogenase

The pixel-based classifier successfully detected the nuclei for measurement of JAM-A intensity in the surrounding cytoplasm/membrane (Fig. 2a, b). The quantitative analysis supported the qualitative observations illustrating both inter- and intratumoral variations in JAM-A intensity (Fig. 2c–f and Online Resource 1). In the RSD cohort, JAM-A intensity was higher in grade II (p < 0.001), grade III (p < 0.001), and grade IV gliomas (p < 0.001) compared to normal brain tissue (Fig. 2c). Further, JAM-A intensity was significantly higher in grade IV tumors than grade II tumors (p < 0.05). No significant differences in JAM-A intensity were seen among the different glioma subtypes (Fig. 2d). In the OUH astrocytoma cohort, no difference was observed in JAM-A levels between DAs and AAs (Fig. 2e). Similar was found when subdividing the DAs and AAs based on IDH status (Fig. 2f).

To further investigate the association between JAM-A and tumor aggressiveness, we used the TCGA dataset comparing the JAM-A mRNA expression level in primary, secondary, and recurrent GBMs. JAM-A expression was significantly higher in recurrent GBMs than primary GBMs (p < 0.001), while secondary GBMs tended to have a higher expression level than primary GBMs (p > 0.05) (Online Resource 2).

### JAM-A and survival

**Fig. 3:**
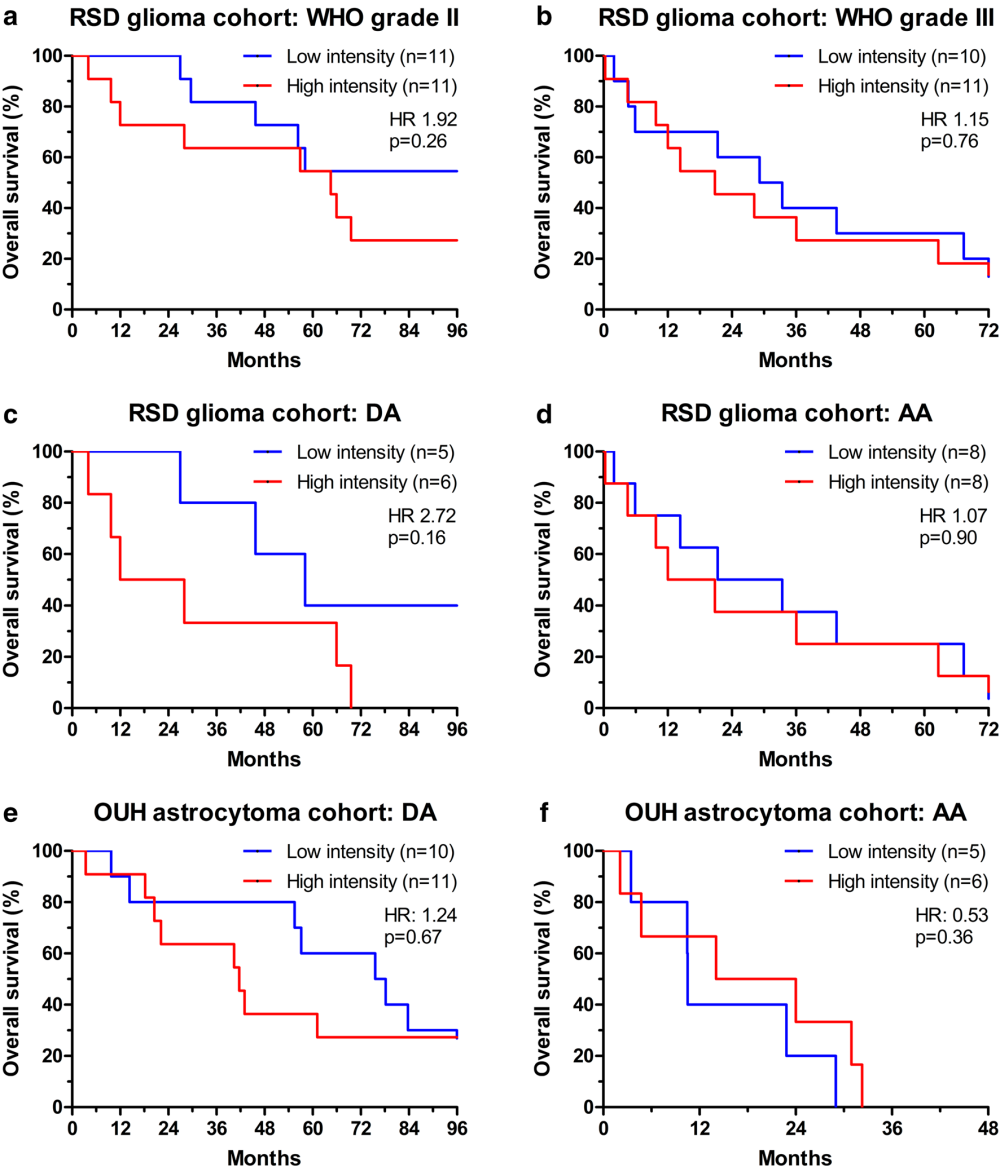
Association between JAM-A intensity and overall survival. Kaplan–Meier curves for patients with **a** WHO grade II and **b** grade III glioma in the RSD glioma cohort. Kaplan–Meier curves for patients with **c** DA and **d** AA in the RSD glioma cohort. Kaplan–Meier curves for patients with **e** DA and **f** AA in the OUH astrocytoma cohort. *AA* anaplastic astrocytoma, *DA* diffuse astrocytoma, *OUH* Odense University Hospital, *RSD* Region of Southern Denmark

In the RSD glioma cohort, JAM-A intensity was not associated with OS in grade II (HR 1.92; 95% CI 0.63–5.87; p = 0.26) (Fig. 3a) or grade III tumors (HR 1.15; 95% CI 0.46–2.85; p = 0.76) (Fig. 3b). Looking only at patients with DA, high JAM-A intensity tended to correlate with shorter survival when divided at the median (HR 2.72; 95% CI 0.67–11.01; p = 0.16) (Fig. 3c). In patients with AA, JAM-A did not correlate with survival when dichotomized at the median (HR 1.07; 95% CI 0.38–2.97; p = 0.90) (Fig. 3d).

In the OUH astrocytoma cohort, no difference in survival was seen for patients with DA (HR 1.24; 95% CI 0.46–3.35; p = 0.67) (Fig. 3e) or AA (HR 0.53; 95% CI 0.14–2.03; p = 0.36) (Fig. 3f) when dichotomized at the median.

### Co-localization of JAM-A and other markers

**Fig. 4:**
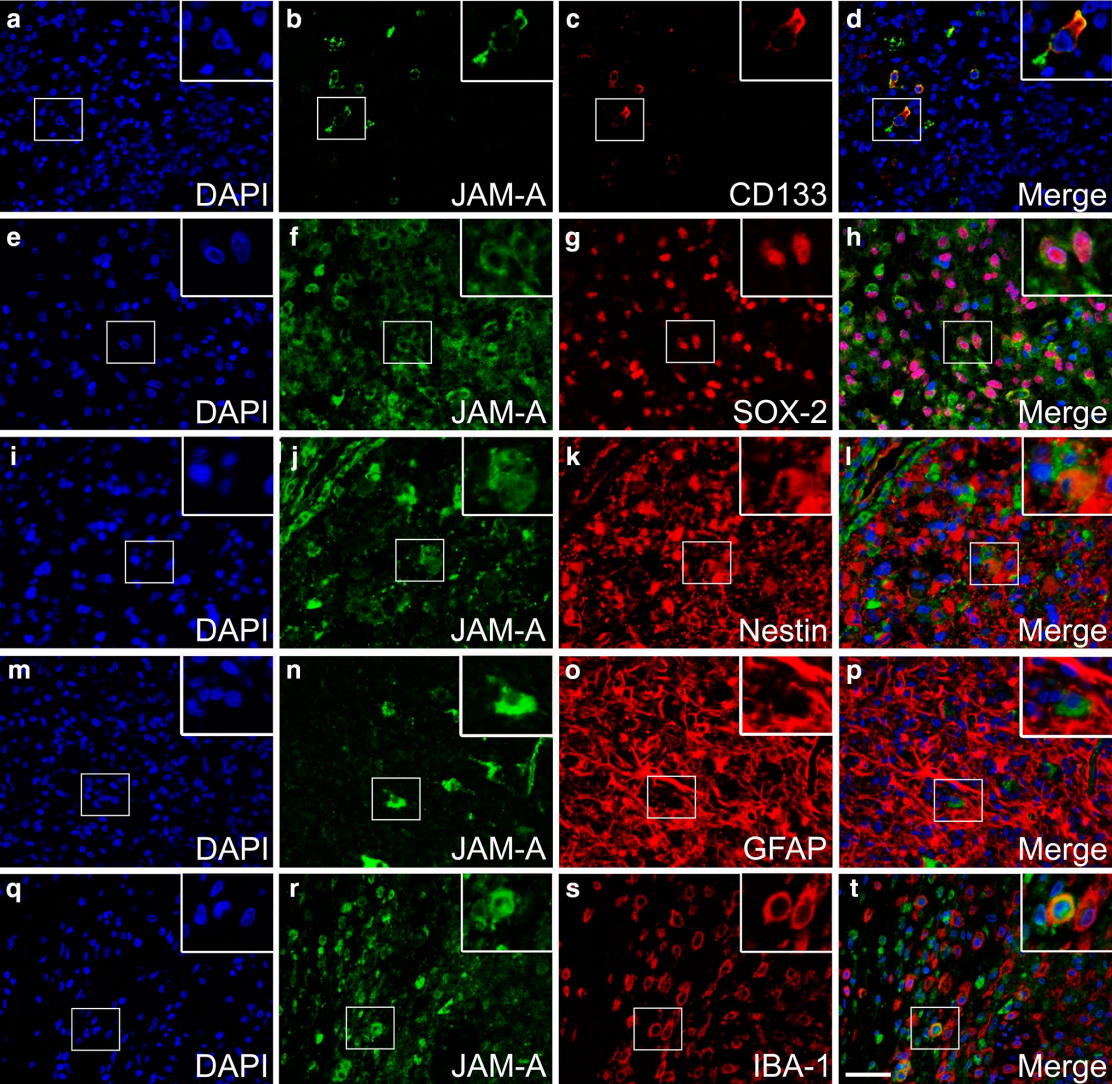
Co-expression of JAM-A in glioblastomas using immunofluorescence. **a**–**d** JAM-A/CD133 co-expression was seen, and **e**–**h** JAM-A/SOX2 co-expression was observed in some areas of the glioblastomas. **i**–**l** Most tumor cells did not express both JAM-A and nestin. **m**–**p** JAM-A+ cells rarely expressed GFAP. **q**–**t** The microglial/macrophage marker IBA-1 was expressed by a few JAM-A+ cells. *Scale bar* 50 µm

Using double-immunofluorescence, we found that a few JAM-A+ cells co-expressed CD133 (Fig. 4a–d). Some cells expressed both SOX-2 and JAM-A (Fig. 4e–h). However, many SOX-2+ cells did not express JAM-A. Nestin (Fig. 4i–l) and GFAP (Fig. 4m–p) rarely co-localized with JAM-A. The microglial/macrophage marker IBA-1 co-localized with a few JAM-A+ in GBMs, but most IBA-1+ cells were did not express JAM-A (Fig. 4q–t).

## Discussion

To our knowledge, this is the first study investigating JAM-A protein expression in grade II-III gliomas. We found that JAM-A was expressed in all gliomas included in this study. The JAM-A intensity increased with malignancy grade, while its prognostic value was limited.

In normal brain tissue, we observed some JAM-A+ cells including sub- and ependymal cells, neurons in the neocortex, as well as a few cells in the white matter possibly of microglial [45] or oligodendroglial [46] origin. Endothelial cells were also shown to express JAM-A in both normal [45, 47] and malignant brain tissue.

JAM-A was expressed in both the cytoplasm and membrane of tumor cells. In normal epithelial tissues, JAM-A has usually been reported to be localized to the cellular membranes [12]. However, in normal colon tissue JAM-A shows a distinct membrane expression, while it is also localized in the cytoplasm in colon cancer [48]. During our protocol optimization of the JAM-A antibody, a tissue microarray containing both normal and cancer tissues was stained. We found that e.g., normal liver tissue and breast carcinomas had a distinct membrane staining (Online Resource 1, shown for breast carcinomas), suggesting that the cytoplasmic staining observed in gliomas is a true part of the staining pattern. This change in cellular location could be due to the influence of the tumor micro-environment. A shift in the intercellular junction from the inter-endothelial to apical surface has been noticed in brain endothelial cells when exposed to cytokines [47]. Gliomas are infiltrated with non-tumor cells such as leukocytes and microglia both secreting cytokines. Possibly, the cytoplasmic staining of JAM-A could also reflect a higher turnover of the protein. One function of JAM-A is to stabilize integrins thereby facilitating adhesion between cells [16]. This may be in line with a high expression and an important function in tumor-initiating cells, which to some extent is supported by our finding that JAM-A co-localized with especially CD133 and to a lesser degree with SOX-2, while JAM-A seemed to only co-localize with GFAP and nestin to a minor degree. Our double-immunofluorescence results could suggest that JAM-A expression may decrease when the tumor cells become more differentiated. However, this needs to be investigated further performing systematic analyses on the double-immunofluorescence stainings.

We have previously identified a prognostic value of JAM-A protein in GBMs [14]. In the present study, JAM-A was not a prognostic marker in grade II and III in the RSD glioma cohort, but the number of patients was small. We performed an analysis on the astrocytomas alone and noticed that, although not significant, the prognostic impact of JAM-A increased in DAs. We therefore hypothesized that the association with survival was more pronounced in DAs and AAs than in grade II and III gliomas in general. Thus, the OUH cohort with 21 DAs and 11 AAs was stained, but no significant association with OS was observed. Interestingly, high levels of JAM-A tended to associate with longer survival in patients with AA in the OUH astrocytoma cohort, while this was not the case in RSD glioma cohort; this could be due to the uneven distribution of IDH mutated tumors, as most AAs in the RSD cohort had mutations in IDH, while most AAs in OUH cohort were IDH wildtype.

The prognostic role of JAM-A is debated in other cancer types [49]. High expression is associated with poor outcome in lung and nasopharyngeal carcinoma [20, 21], but in kidney, pancreatic, and gastric cancer high expression of JAM-A is associated with better prognosis [22–24]. In the present study, some IBA-1+ microglia/macrophages also expressed JAM-A as expected from the observed cellular morphology [10, 47], but the extent of co-expression was limited confirming previous findings [45]. Whether this may influence results obtained in other studies is unknown, but JAM-A labeling of non-tumor cells may be important in some cancers. The role of JAM-A in cancer biology thus seems complex.

A strength in our study is the use of a software-based quantitative approach that is more objective than observer-based scoring [50]. This approach prevents intra-observer variation, and as an advantage JAM-A intensity is measured on a continuous scale.

In conclusion, we demonstrated that JAM-A expression is higher in GBMs than in low-grade gliomas and that JAM-A co-localizes with recognized BTIC markers. Further, results from the TCGA showed that JAM-A mRNA levels were higher in recurrent GBMs compared to primary. Together with the earlier findings showing that JAM-A is an independent prognostic factor in GBMs, the results suggest a close association between JAM-A and glioma aggressiveness. No association was found between JAM-A expression and OS in grade II and III gliomas. Further research is needed to determine the function and clinical impact of JAM-A in gliomas.

## Electronic supplementary material

Below is the link to the electronic supplementary material.

Supplementary material 1 (PDF 458 KB)

Supplementary material 2 (PDF 146 KB)
