# Supplementary material for: Expression and prognostic value of JAM-A in gliomas
Source: J Neurooncol. 2017 Jul 4;135(1):107–17. doi: 10.1007/s11060-017-2555-0 (PMC5658466; doi:10.1007/s11060-017-2555-0)
Supplement: Supplementary file 1 — Supplementary material 1 (PDF 458 KB) [file 11060_2017_2555_MOESM1_ESM.pdf]

Expression and prognostic value of JAM-A in gliomas

Ann Mari Rosager[1,2*], Mia D. Sørensen [1,2*], Rikke H. Dahlrot[3], Henning B. Boldt[1], Steinbjørn Hansen[2,3], Justin D. Lathia[4], Bjarne W. Kristensen[1,2]

* These authors contributed equally to this work

1. Department of Pathology, Odense University Hospital, Odense, Denmark
2. Department of Clinical Research, University of Southern Denmark, Odense, Denmark
3. Department of Oncology, Odense University Hospital, Odense, Denmark
4. Department of Cellular and Molecular Medicine, Lerner Research Institute, Cleveland, USA

Corresponding author: Email: mia.soerensen@rsyd.dk

Online Resource 1, Journal of Neuro-Oncology

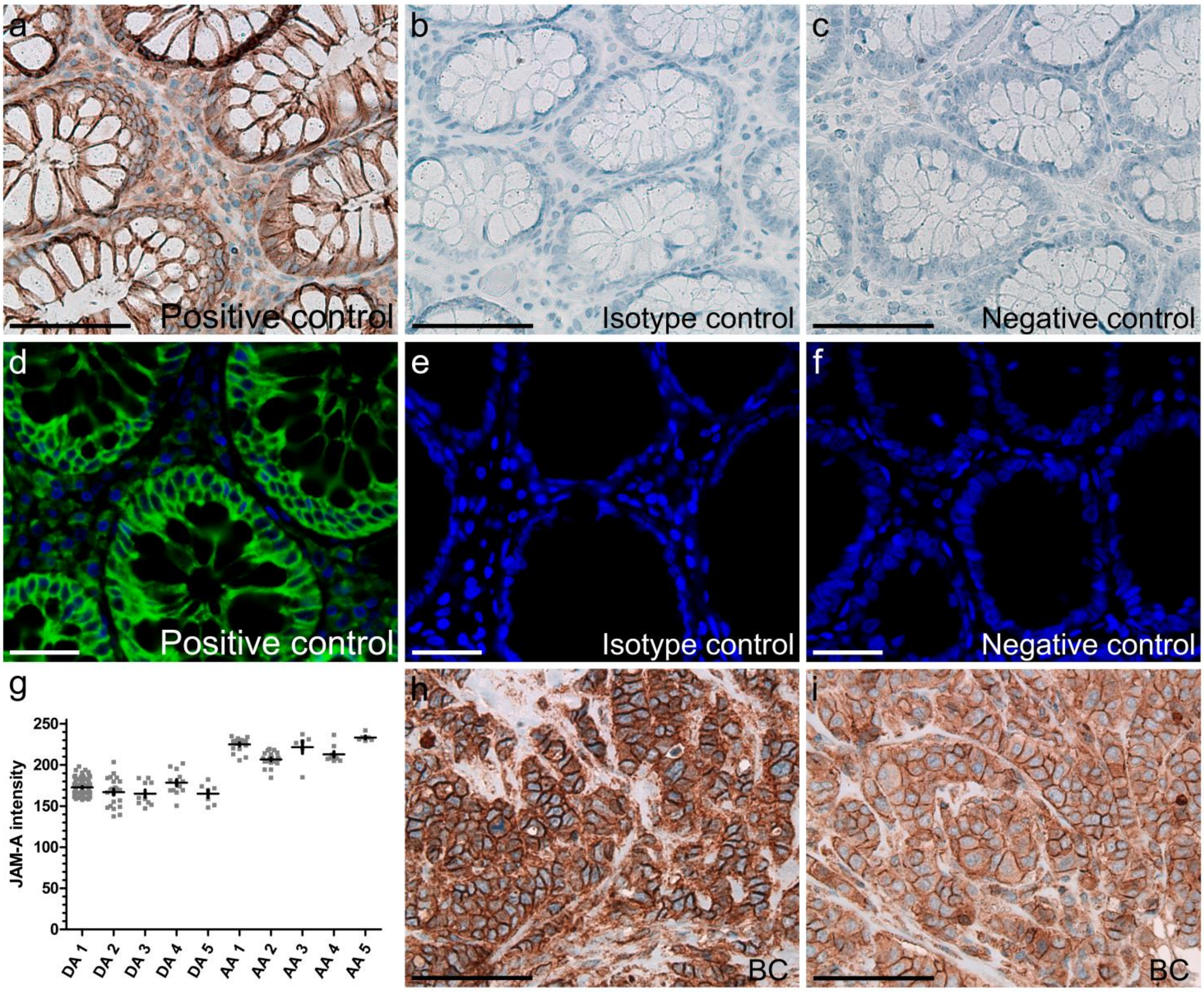

Online Resource 1 JAM-A staining controls and JAM-A intensity in ten gliomas
To demonstrate the staining specificity of the JAM-A antibody used in immunohistochemical (**a-c**) and fluorescence (**d-f**) staining protocols, **a and d** the positive controls, **b and e** isotype controls, and **c and f** negative controls are shown for normal colon tissue. **g** To illustrate the intra- and inter-tumoral heterogeneity of JAM-A expression in gliomas, a scatter plot of JAM-A intensity range is shown for five diffuse astrocytomas (DA1-5) and five anaplastic astrocytomas (AA1-5). All intratumoral measurements and the corresponding mean value (horizontal line) and standard error of the mean (vertical lines) are illustrated for each tumor. **h and i** In contrast to the combined cytoplasmic/membrane staining pattern of JAM-A in gliomas, breast carcinomas (BC) showed a distinct membrane expression. Scale bar: 100 µm (a-c, h-i) and 50 µm (d-f).
