# Supplementary material for: Expression and prognostic value of JAM-A in gliomas
Source: J Neurooncol. 2017 Jul 4;135(1):107–17. doi: 10.1007/s11060-017-2555-0 (PMC5658466; doi:10.1007/s11060-017-2555-0)
Supplement: Supplementary file 2 — Supplementary material 2 (PDF 146 KB) [file 11060_2017_2555_MOESM2_ESM.pdf]

Expression and prognostic value of JAM-A in gliomas

Ann Mari Rosager[1,2*], Mia D. Sørensen [1,2*], Rikke H. Dahlrot[3], Henning B. Boldt[1], Steinbjørn Hansen[2,3], Justin D. Lathia[4], Bjarne W. Kristensen[1,2]

* These authors contributed equally to this work

1. Department of Pathology, Odense University Hospital, Odense, Denmark
2. Department of Clinical Research, University of Southern Denmark, Odense, Denmark
3. Department of Oncology, Odense University Hospital, Odense, Denmark
4. Department of Cellular and Molecular Medicine, Lerner Research Institute, Cleveland, USA

Corresponding author: Email: mia.soerensen@rsyd.dk

Online Resource 2, Journal of Neuro-Oncology

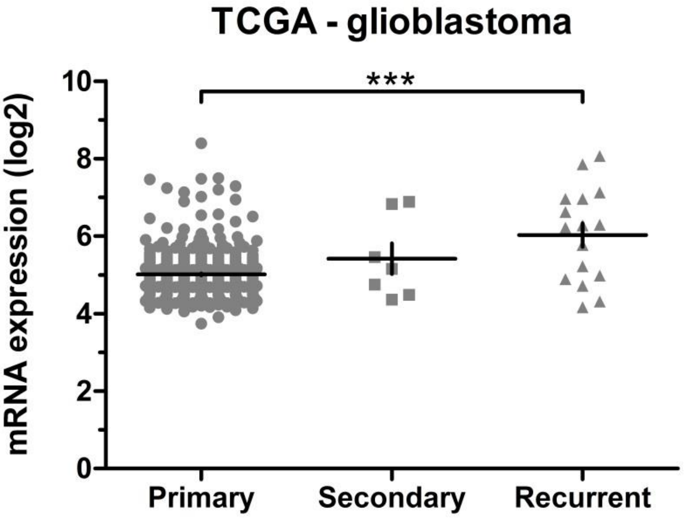

Online Resource 2 JAM-A mRNA expression in glioblastomas
The JAM-A mRNA levels were significantly higher in recurrent glioblastomas (n=16) compared to primary glioblastomas (n=497) when investigated in the Cancer Genome Atlas (TCGA) dataset. The JAM-A expression level in secondary glioblastoma (n=7) did not differ significantly from the expression level in recurrent glioblastomas, but tended to be higher than the mRNA level in primary glioblastomas.
